# Can Andean medicine coexist with biomedical healthcare? A comparison of two rural communities in Peru and Bolivia

**DOI:** 10.1186/1746-4269-8-26

**Published:** 2012-07-24

**Authors:** Sarah-Lan Mathez-Stiefel, Ina Vandebroek, Stephan Rist

**Affiliations:** 1 Centre for Development and Environment, University of Bern, Hallerstrasse 10, 3012, Berne, Switzerland; 2 Institute of Economic Botany, The New York Botanical Garden, 2900 Southern Boulevard, Bronx, NY, 10458, USA; 3 Centre for Development and Environment, University of Bern, Hallerstrasse 10, 3012, Berne, Switzerland

**Keywords:** Biomedicine, Traditional medicine, Self-treatment, Pharmaceuticals, Natural remedies, Culture-bound illnesses, Bolivia, Peru

## Abstract

**Background:**

It is commonly assumed that indigenous medical systems remain strong in developing countries because biomedicine is physically inaccessible or financially not affordable. This paper compares the health-seeking behavior of households from rural Andean communities at a Peruvian and a Bolivian study site. The main research question was whether the increased presence of biomedicine led to a displacement of Andean indigenous medical practices or to coexistence of the two healing traditions.

**Methodology:**

Open-ended interviews and free listing exercises were conducted between June 2006 and December 2008 with 18 households at each study site. Qualitative identification of households’ therapeutic strategies and use of remedies was carried out by means of content analysis of interview transcriptions and inductive interference. Furthermore, a quantitative assessment of the incidence of culture-bound illnesses in local ethnobiological inventories was performed.

**Results:**

Our findings indicate that the health-seeking behavior of the Andean households in this study is independent of the degree of availability of biomedical facilities in terms of quality of services provided, physical accessibility, and financial affordability, except for specific practices such as childbirth. Preference for natural remedies over pharmaceuticals coexists with biomedical healthcare that is both accessible and affordable. Furthermore, our results show that greater access to biomedicine does not lead to less prevalence of Andean indigenous medical knowledge, as represented by the levels of knowledge about culture-bound illnesses.

**Conclusions:**

The take-home lesson for health policy-makers from this study is that the main obstacle to use of biomedicine in resource-poor rural areas might not be infrastructural or economic alone. Rather, it may lie in lack of sufficient recognition by biomedical practitioners of the value and importance of indigenous medical systems. We propose that the implementation of health care in indigenous communities be designed as a process of joint development of complementary knowledge and practices from indigenous and biomedical health traditions.

## Background

The developing world’s population relies heavily on indigenous medicine to meet its health care needs. In some developing countries, up to 80% of the population uses indigenous medicine for primary health care [1]. The World Health Organization and the World Bank explain the relationship between poverty and poor health as follows:

"*“When formal health care services are unavailable, people turn to traditional medicine. In most cases, they say that they would rather be treated by modern health care providers, but often traditional services are all they can access or afford”*([2], p.24)."

This quotation summarizes a commonly held assumption among health policy-makers and researchers since the beginning of the 1980s, namely that indigenous medical systems remain strong in developing countries because formal health care is physically inaccessible or not affordable [3,4]. Young and Garro [5], in a comparative study of two Mexican communities that share the same ethnomedical beliefs, observed that inhabitants from the community with better access to biomedicine consulted a physician nearly twice as much as people from the other community. The corollary of this assumption is that increased access to biomedicine displaces indigenous medical knowledge and practices through a process of “medical hegemony” [6], a view supported by several authors [7-10].

In the present paper, we investigate this assumption by comparing the health-seeking behavior of households from rural Andean Quechua communities in Peru and Bolivia. The main research question was whether the increased presence of biomedicine led to a displacement of Andean medicine or to coexistence of indigenous and biomedical health care. Our hypothesis was that at the Peruvian field site, where biomedical facilities are more accessible and affordable than at the Bolivian site, the use of Andean medicine would be at a lower level. We combined a qualitative research approach to identify and analyze households’ therapeutic strategies and use of remedies with quantitative assessment of the incidence of culture-bound illnesses in local ethnobiological inventories.

Medical pluralism refers to the coexistence of various medical traditions. Baer [11] describes a widely used model developed by Chrisman and Kleinman [12] that distinguishes three overlapping sectors in pluralistic health care systems: 1) the popular sector (health care provided by the sick persons themselves, their families, social networks, and communities); 2) the folk sector (health care provided by traditional healers, including herbalists, bonesetters, midwives, mediums, and magicians); and 3) the professional sector (health care provided by practitioners and institutions in biomedicine and professionalized heterodox medical systems, such as Chinese, Ayurvedic and Unani medicine). Researchers working in the Andes have recognized the coexistence of these three types of medical traditions [13,14]. We will hereafter use the terms “indigenous” or “Andean” medicine to refer to the popular and folk sectors and the terms “biomedicine” and “formal health care” to refer to the professional sector as defined by Chrisman and Kleinman [12]. Within Andean medicine, we further distinguish self-treatment (popular sector) from recourse to healers (folk sector).

Historically, scholars who study medical pluralism have often considered these different medical systems as closed or even competing domains [15,16]. However, Crandon-Malamud [14] suggests that this view is the result of artificial boundaries set up by researchers for analytical purposes, and that exchanges between systems may be ubiquitous. Some authors stress that indigenous medicine and biomedicine might be part of the same cognitive system, characterized by the integration and syncretism of the medical beliefs, knowledge and practices of these two healing traditions [17-23]. Other researchers affirm that complementarities can exist between these contrasting medical systems, as exemplified by the multiple use of these systems by sick persons or active collaboration between practitioners of both types of medicine [24-29]. Nonetheless, one must also consider “the unequal global power relations involved in the creation and use of particular health care systems in specific places by different groups of people” ([10], p.305). This is especially true when looking at the interactions between biomedicine and indigenous medical systems, given the overall dominance of the former as a global and state-supported apparatus over the latter as a local cultural model [6,9-11,30,31].

### Research setting and study sites

This study was part of the research component of BioAndes, an international cooperation program that aimed to contribute to the conservation of biocultural diversity in the Bolivian, Peruvian, and Ecuadorian Andes [32]. Formal agreements were signed between the program’s implementing agencies and their governmental counterparts at the national and municipal levels. The study was carried out at the Institute of Geography of the University of Bern, which does not have an ethical review board. However, it was approved by the boards of both the Swiss National Centre of Research North–South and the Swiss Commission for Research Partnerships with Developing Countries (KFPE). Both institutions subscribe to the KFPE’s Eleven Principles for Transboundary Research Partnerships [33].

**Figure 1:**
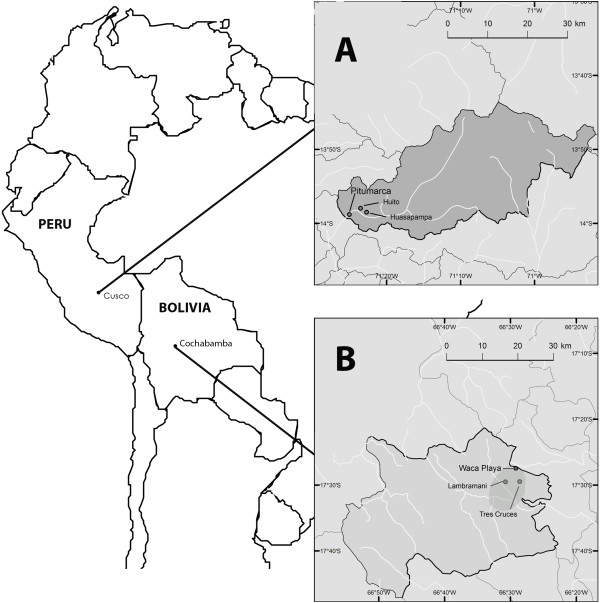
**Map of the study sites [**[35]**].** Research was conducted in Pitumarca District in the Department of Cusco, Peru (**A**) and in Waca Playa Subcentral in the Province of Tapacari, Department of Cochabamba, Bolivia (**B**).

Research was conducted by the first author between June 2006 and April 2010 in two of the seven field areas of the BioAndes program: the rural communities of Waca Playa (17°27'30"S, 66°29'21"W) in Tapacarí Province, Cochabamba Department, Bolivia, and Pitumarca (13°58’48”-S; 71°25’W) in Canchis Province, Cusco Department, Peru (Figure 1). Although located in two different countries, these study sites were selected for their historical, sociocultural, and ecological similarities despite differing availability of formal health care services, as shown in Table 1.

**Table 1:** Variables of human development, health, and availability of biomedical facilities in Waca Playa and Pitumarca

	**Waca Playa (Bolivia)**	**Pitumarca (Peru)**
Human Development Index	0.36 ^a^	0.51 ^b^
Life expectancy (years)	52.4 ^a^	70.0 ^b^
National social health security	SUMI: coverage for pregnant women and children under age 5	SIS: coverage for all adult population living in poverty or extreme poverty
Community biomedical facilities	Rudimentary medical post with one auxiliary nurse provides ambulatory health care to 20 villages. Approximately one visit by auxiliary nurse every two months to each village.^c^	Health center with 11 health professionals, including one medical doctor. One visit by a nurse per month to each village. 4 to 6 health workers trained in each village. ^c^
Distance from community to villages where households were interviewed (km)	5 to 9 ^c^	4 to 5 ^c^
Distance from community to nearest hospital (km)	approximately 40 (dirt road) ^c^	35 (paved road) ^c^
Beds per inhabitant (number)	0.66 ^d^	0.84 ^e^

^a^UNDP. Human Development Index. 2001. Bolivia. (Data from Tapacarí Province).

^b^UNDP. Human Development Index. 2007. Peru.

^c^Own observations and interviews with health professionals in the field.

^d^INE. 2001. Censo de Población y Vivienda. 2001. Bolivia. (Data from Tapacarí Province).

^e^INEI. Compendio Estadístico Departamental 1995-1996. Departamento de Cusco. Peru. (Data from Canchis Province).

**Figure 2:**
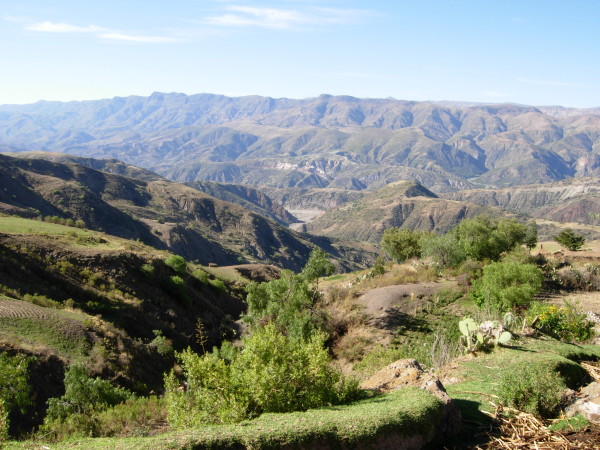
View from the village of Tres Cruces in Waca Playa Subcentral, Bolivia.

**Figure 3:**
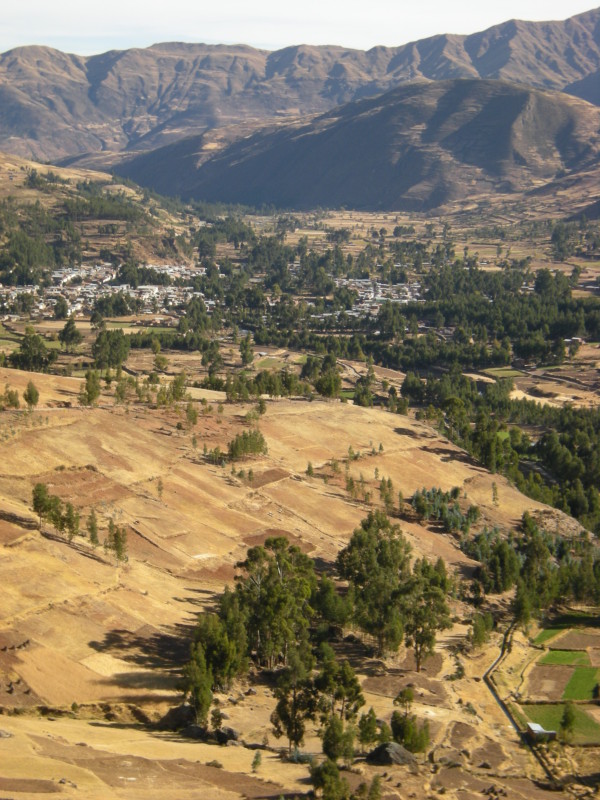
**Valley of Pitumarca, Peru.** The town of Pitumarca can be seen in the background.

Waca Playa is a rural subcentral in the Bolivian Eastern Cordillera, 65 km east of the city of Cochabamba. Its capital is located at 3,950 m.a.s.l., on the top of a hill that overhangs the territory of five peasant villages (Figure 2). The site belongs to the “Central Andean Puna” (montane grassland and shrubland biome) and “Bolivian Montane Dry Forests” (tropical and subtropical dry broadleaf forests biome) global ecoregions [34]. Pitumarca is a rural district in the Southern Peruvian Andes, 87 km south-west of the city of Cusco. The town is situated at 3,580 m.a.s.l., at the bottom of the Pitumarca Valley (Figure 3). The site corresponds to the “Central Andean Wet Puna” ecoregion (montane grassland and shrubland biome) [34]. Both sites display a similar anthropogenic landscape, composed of a mosaic of small cultivated parcels, remnant patches of native forests (e.g. *Polylepis* spp.) and exotic plantations (e.g. *Eucalyptus* spp. and *Pinus* spp.), rivers, rocks, and grasslands.

Both study sites are inhabited by indigenous Quechua-speaking farmers who gained community property rights over their territory after the agrarian reforms (in 1953 in Bolivia and from 1968 to 1975 in Peru). At the village level, authorities are elected from amongst the households on a rotational basis. Decisions are taken at monthly assemblies, where all the village’s households are represented. The population of both sites is mainly engaged in subsistence farming with the growing of grains (e.g. maize, wheat, quinoa, barley, oats), many varieties of potatoes and other Andean tubers (e.g. *Ullucus tuberosus* Caldas and *Oxalis* spp.), vegetables and fruits, and the herding of livestock (sheep, goats, cows, and also lamas and alpacas in Pitumarca’s highlands). The surplus production is sold at local and regional markets. Local livelihoods are supplemented by temporal migration to the Amazon lowlands or to the urban centers for remunerated work. In addition, some households are also engaged in commercial activities [35].

**Figure 4:**
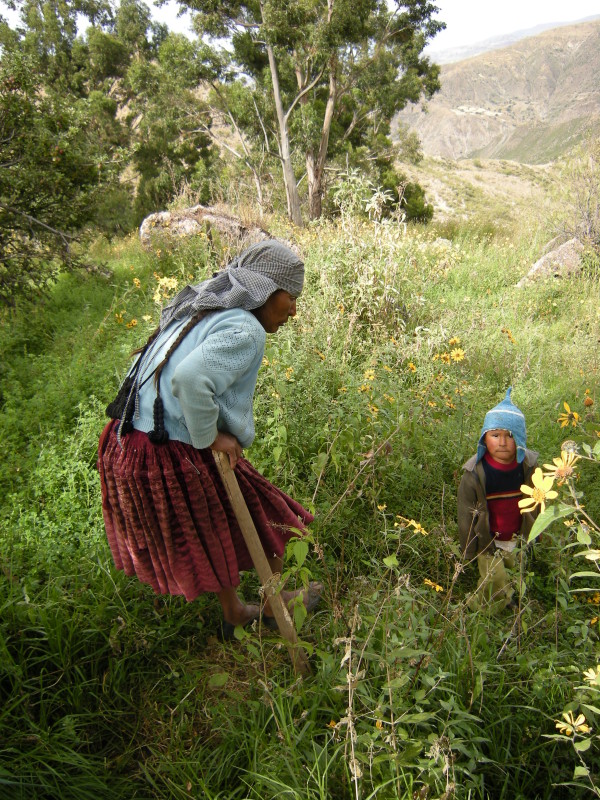
Participant in the study collecting medicinal plants around her house in the village of Lambramani, Waca Playa, Bolivia.

**Figure 5:**
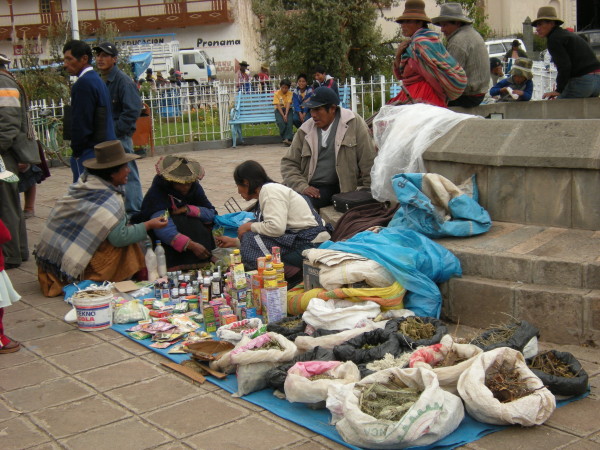
Medicinal plants sold at the weekly market of Pitumarca, Peru.

The most common health problems found at both sites are respiratory, gastrointestinal, parasitic, infectious, skin diseases, and labor complications [36,37]. Andean medicine is an important health resource for the local population; it is performed by laypeople themselves, who are highly knowledgeable about medicinal plants and animals, and by specialists such as healers, bonesetters, and midwives [36,37]. Most natural remedies are collected locally, but others grow outside the study sites and are bought at local markets (Figures 4 and 5).

**Figure 6:**
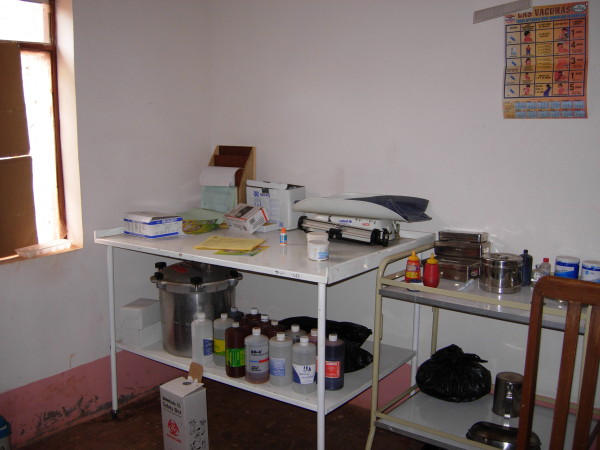
Waca Playa’s health post, Bolivia.

From Table 1, it is clear that formal health care is better in Pitumarca than in Waca Playa, both in terms of the quality of services provided and physical access to the nearest hospital. In Waca Playa a small health post with an auxiliary nurse was established only in 1995 (Figure 6). In contrast, Pitumarca has had a health center since the 1960s where eleven health professionals now operate (one medical doctor, two obstetricians, two nurses, and six auxiliary nurses). Furthermore, whereas there are no health workers based in Waca Playa’s villages, in Pitumarca village members are recruited by the health center as volunteer health workers. They receive basic training once a month in nutrition, hygiene, breastfeeding, and prevention of common afflictions such as parasitic illnesses, pneumonias, and diarrhea. Their main task is to ensure that sick villagers, young children and pregnant women go to the health center for treatment, vaccinations, or routine check-ups. Table 1 also shows that biomedical health services are more affordable to the local population in Pitumarca (Peru) than in Waca Playa (Bolivia). Social health security coverage is better in Pitumarca. In Bolivia, the SUMI (*Seguro Universal Materno Infantil*) was created by Law 2426 in 2002 and provides coverage for pregnant women until six months after childbirth and for children up to age five. In Peru, the SIS (*Seguro Integral de Salud*) was created by Law 27675 in 2002, resulting from the fusion of the SMI (*Seguro Materno Infantil*), which provided coverage for pregnant women and children up to the age of five living in conditions of poverty or extreme poverty, and the SEG (*Seguro Escolar Gratuito*), which gave free health care to students in state schools under age 17 . In 2005, Peruvian Law 28588 extended SIS coverage to all adults, regardless of age, living in conditions of poverty or extreme poverty – which applies to the population of Pitumarca. The United Nations Development Program’s Human Development Index is also higher in Pitumarca than in Waca Playa, which implies that Pitumarquinos have an overall higher income per capita, better education, and longer life expectancy.

## Methodology

### Data collection

Data for this study were collected between March 2007 and May 2008 in Waca Playa and between June and December 2008 in Pitumarca. Prior informed consent was obtained orally from community representatives and from each person interviewed prior to data collection. Open-ended interviews and free listing exercises were conducted with 18 households at each study site. In addition, expert interviews were conducted with representatives from the formal (one in Waca Playa and two in Pitumarca) and indigenous (two in Waca Playa and two in Pitumarca) health sectors at both study sites, as well as with three health workers from Pitumarca from among the participating households. Interviews were conducted in Quechua, with the help of a native Quechua-speaking translator, and/or in Spanish when the interviewees were also fluent in this language. They were recorded, translated into Spanish where needed, and transcribed for further analysis.

All the participating households from Waca Playa and ten from Pitumarca were selected at village assemblies, in order to represent young (20 to 34 years old), middle-aged (35 to 49 years old), and elderly (50 years old and above) households. The remaining eight participating households from Pitumarca volunteered themselves. Participants represented the villages of Tres Cruces (nine households, 18% of all households) and Lambramani (nine households, 23% of all households) in Waca Playa and the villages of Huasapampa (ten households, 16% of all households) and Huito (eight households, 13% of all households) in Pitumarca. According to the age of the main interviewee in each household, two participants from Waca Playa were young, seven were middle-aged, and nine were elderly. In Pitumarca, six participants were young, seven middle-aged, and five elderly. Most participants from Waca Playa had no formal education or had reached the primary level. Only one participant had more than six years of education. In Pitumarca, thirteen participants had no formal education or less than six years of education, while five participants had seven or more years of formal education. Interviews took place during three to six visits to each household with the husband and/or the wife. Each interview lasted approximately one to three hours. Information was also occasionally cross-checked by means of additional interviews with the adult children. Data were collected at the household level because preliminary field work showed that illness and well-being were family issues. Decisions regarding health-seeking options were usually taken by the sick person (or his/her parents in the case of children) after discussion with the other household members, and sometimes also with the extended family (parents, siblings, etc. from other household units).

Health-seeking behavior of households was investigated by means of open-ended interviews. Households were invited to explain their concepts of health and illness, describe the more common illnesses that occurred in their family, their symptoms and etiology, and explain their health-seeking strategies. Preliminary informal interviews at the study sites allowed us to identify three prevalent strategies in the communities: 1) self-treatment with natural remedies (medicinal plants, animals, and minerals); 2) consultation with an Andean healer; 3) visits to the health center. During the open-ended interviews, households were asked which of these three strategies they chose as a first option, and which they adopted as a second option if the first strategy did not work. They were also asked whether they used pharmaceutical or natural remedies (including plants, minerals and animals), and the types of remedies they preferred. In addition, households were invited to explain thoroughly their health-seeking choices and preferences and provide examples based on their own experience.

Free listing was used to assess the incidence of “culture-bound illnesses” (CBIs, hereafter) at each study site. CBIs, also called “culture-bound syndromes” or “folk illnesses”, can be defined as “illnesses that are bounded according to cultural systems and are often not directly translatable into biomedical terms” ([38], p. 282). According to this definition, all illnesses could be considered “culture-bound” from an anthropological perspective. Nonetheless, preliminary field work at the study sites revealed that local people clearly distinguished a set of illnesses considered specific to Andean culture from the other ailments that affected them. Households were asked first to list all the natural remedies (plants, animals, minerals) that they knew and/or used, as well as all the illnesses that were treated with each of these remedies. In addition, they were asked to describe these illnesses in detail.

Health experts were interviewed individually about their professional activities, their views on biomedicine and Andean medicine, and their perception of the health situation and the health-seeking behavior of the local population. Finally, they were asked to assess changes that may have occurred in the population’s therapeutic strategies in recent decades.

### Data analysis

Answers from participating households about their health-seeking strategies and their use and preference of types of remedies were summed at each study site. The proportions of households that expressed a preference for pharmaceutical over natural remedies were compared by means of the z-test for comparison of proportions with Yates correction. Next, interview transcriptions were analyzed qualitatively to obtain in-depth understanding of these strategies and preferences, according to participants’ own perspectives. As suggested by grounded theory [39], content analysis and inductive inference were applied to interpret the discourses by which the participants imbue social reality with meaning. Content analysis consisted in fragmenting the interview transcriptions into units of information for their subsequent coding and categorization, and inductive inference consisted in formulating generalizations based on the observations and prior theoretical conceptions [40].

Inventories of plants, animals, and minerals and their medicinal uses were compiled for each study site based on the results from the household free listing exercises. These ethnobiological inventories were the basis for assembling a list of illnesses treated with natural remedies at each site. CBIs were then identified from these lists according to the participants’ description of illnesses, when the symptoms and/or etiology could be considered specifically part of Andean culture. These included health problems with multiple symptoms, including reference to emotional, physical and spiritual health, reference to social or spiritual causes, and reference to the humoral hot/cold classification of diseases widespread in Latin American medical systems. The incidence of CBIs at each study site was calculated as the percentage of total responses on medicinal use given by participating households during the free listing exercises. A response is a report on the use of remedy X to treat illness Y by household Z. The z-test for comparison of proportions with Yates correction was applied to compare the results for the incidence of CBIs from the two study sites.

### Methodological limitations of the study

This work should be understood as an exploratory study that aimed to enrich a mainly qualitative approach with quantitative analyses. The main methodological limitation of this mixed approach was the reduced sample size (18 households at each site). A total of 36 households was the largest number of participants we were able to collaborate with during the 22 months of data collection, which included long open-ended interviews, informal exchanges, and participation in the villagers’ daily activities. We believe that the ethnographic data obtained through this approach combined with qualitative analysis allowed for strengthening of the observations drawn from the results.

## Results and discussion

### Household health-seeking strategies

When faced with illness, almost all participating households from the two study sites first try to cure themselves through self-treatment with natural remedies (18 and 17 of the households from Waca Playa and Pitumarca respectively). The only household from Pitumarca that goes directly to the health center explained this behavior by its limited knowledge about medicinal plants, which made it dependent on formal health care. Our ethnographic data show that being able to rely on one’s own medicinal plant knowledge and local natural resources for self-treatment was highly valorized by all participants at both study sites. The other treatment options (biomedical doctors and Andean healers) were often met with suspicion and critical evaluation, as illustrated by the following quotation:

"*“In vain we go to the doctor; he cannot even cure us of a stomach ache. Others also go to the healers, but I think that some of them are thieves because they do not cure us either. (…) Only our plants are better for us; we take our plants and we are immediately healed.” (Waca Playa-Household 9, 04/03/2008)*"

**Figure 7:**
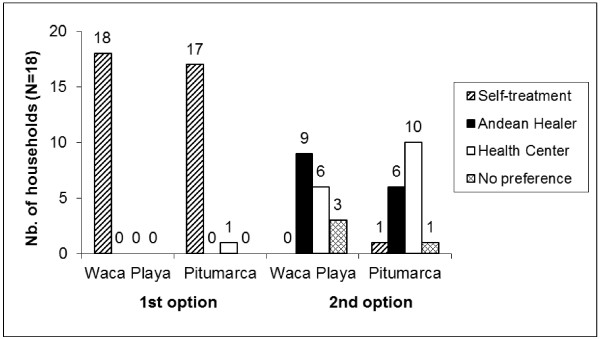
Households’ health-seeking strategies as first and second options in Waca Playa (Bolivia) and Pitumarca (Peru).

If this first health-seeking strategy does not lead to a healthy outcome, then participants either consult a healer or go to the nearest medical post. The number of participating households opting for each of these therapeutic strategies as first and second options at each study site is shown in Figure 7.

 As a second health-seeking option, more households from Waca Playa consulted a healer, while more households from Pitumarca chose to go to the health center, even though these differences are not statistically significant. In Waca Playa, half of all participants preferred consulting an Andean healer as a second option when self-treatment failed. Six of these nine households explained their choice by trust in the healers’ knowledge to heal them or diagnose their ailment and advise on treatment (which might include going to the medical post). Four households stated that going to the medical post was more costly because they would have to pay for medicine, and two affirmed that the medical post was not efficient and did not heal them.

Of the ten Pitumarquino households that chose the health center as a second option, five said that they did not consult healers because there was no need to, as they did not have a serious CBI that required the knowledge of an indigenous specialist, but only minor ailments that could be treated at home with natural remedies or by the medical post staff with pharmaceuticals. Three households said that they went to the health center because they were forced to. They declared that they were severely scolded by the biomedical staff if they did not consult them about illness and might even be blamed for the eventual death of a sick person. Another reason mentioned was lack of good healers in the region (three households). Only two households stated that they did not believe in the healers’ powers, and thus went to the health center if they could not cure themselves with natural remedies.

Three considerations are relevant for better understanding of Andean households’ therapeutic strategies. First, households have a preference for the healer or the health center depending on the illness. At both study sites, households sought the help of a healer in the following cases: “unknown” illnesses that patients were not able to diagnose; illnesses considered “serious” that they were not able to cure; divination and diagnosis by observing *coca* leaves (*Erythroxylum coca* Lam.); CBIs with a spiritual cause, such as *susto* (fright sickness)*, wayra* (bad wind), *rayo*/*qhaha* (lightening) or *hechicería* (witchcraft). Conversely, participants from both study sites said they went to the medical post when they faced the following health problems: illnesses “known” to patients, such as various types of *nanay *(pain) including rheumatism, headache, tooth ache, and stomach ache; fever and common cold; wounds and fractures; pediatric issues (vaccinations and check-ups), and gynecological conditions (contraceptives and pregnancy check-ups, also childbirth in Pitumarca). Only one household from Waca Playa and three from Pitumarca mentioned that they also went to the health post when an illness was considered “serious”, which implied “when our plants are of no use”.

At both study sites, qualitative data showed that illness classification was also strongly related to cultural identity and a sense of belonging to a specific place. Households were clearly aware of the limitations of biomedicine in dealing with CBIs or what are considered “our illnesses”; this view was shared by 14 and 16 of the households in Waca Playa and Pitumarca, respectively. This commonly held belief is exemplified by the following quotation:

"*“I do not know which one [from the medical post or the healer] is best. But for us from the highlands, the post does not have an effect on us and cannot detect our illnesses. So if we need remedies from the highlands the post is not efficient, and if we do not [need remedies from the highlands] we can go to the post and they give us pills and injections. (…)[Illnesses] for the post are illnesses that are only appearing now; only these can be treated by the post.” (Pitumarca-Household 18, 29/09/2008)*"

Participants believed that inhabitants from the highlands suffered from specific illnesses that could only be cured by local natural remedies and the knowledge of Andean healers, whereas the health center could treat new illnesses that came from outside the study site in recent decades by means of pharmaceuticals and injections. Previous studies in the Andes and elsewhere in Latin America refer to the same distinction between ailments and the corresponding choice of treatment made by local people [18,41]. This view corresponds with the earlier concept of “folk dichotomies” in health-seeking behavior (for a history of the concept see [42], cited by [5]). This categorization of illnesses is also consistent with Federico Aguiló’s distinction between “mythical” and “natural” illnesses in Aymara-Quechua concepts of health and illness in Potosi, Bolivia [43]. According to Aguiló, a mythical illness is the result of aggression by a supernatural being proceeding from the hostile environment or the *Pacha Mama* (Mother Earth) that produces a lesion on both the *ajayu* (spirit) and the body. In contrast, a natural illness is aggression by an external agent and a consequent lesion on the peripheral areas of the body, without direct incidence on the *ajayu* ([43], p.11). In her study on fright sickness and soul loss in the Peruvian Andes, Greenway also stressed the relationship between illnesses cured by healers and Quechua identity. She noted that “people were (…) doubtful about the knowledge and ability of non*runa* [non-Quechua] health care practitioners to treat ailments caused by ancestor spirits, evil winds, or other similar causes” and that the realization of “a *despacho *(a burnt sacrificial offering by a traditional healer) serves (…) to reaffirm [the patient’s] (…) identity as a *runa* as opposed to *misti *(white or *mestizo*)” ([41], p. 997).

The second consideration regarding households’ health-seeking strategies is that the different treatment options are not perceived by participants as mutually exclusive, but as complementary. As noted above, there is recognition of the limitations of biomedicine in treating CBIs, but there is also acknowledgment of the contributions that the formal health care system can make to the treatment of other ailments that are mainly physical in origin. This consideration explains the apparent contradiction between feelings of suspicion and trust expressed towards Andean healers. Parallel to the critical perception of both healers and the medical post, there was also awareness that both options might sometimes succeed in reestablishing health and thus are worth a try. While most households trusted the healers more in cases of unknown or serious illnesses, they would still use biomedicine if Andean medicine was not able to cure them. As a result of this perception of the three medical systems as complementary, the health-seeking behavior of households often took the form of a therapeutic itinerary. This pragmatic multiple strategy usually started with self-treatment with natural remedies, then switched to visits to an Andean healer or to the health center, and continued with the other therapeutic option or a return to self-treatment. This behavior was recurrent in the narratives of 12 and 13 of participants from Waca Playa and Pitumarca respectively. A typical way of expressing it follows:

"*“I was told that I had gastritis (…). I was at the hospital in Sicuani for 15 days, but they did not cure me. The doctors told me: 'Go to Cusco [to the hospital] and there they will treat you, if not you will die in less than a week.’(…) Since I did not have money, I came back home and I drank my herbs. (…) I did not pay attention to what they told me. And here there is a yachayniyuc [healer] that reads the coca [Erythroxylum coca* Lam.*]. I made him read the coca, and he told me that it was uraña [bad wind].” (Pitumarca-Household 13, 02/10/2008)*"

This quote shows how individuals make their own therapeutic choices and trials, characterized by changing diagnoses and based on perceived efficacy, knowledge and beliefs, socioeconomic situation, and social networks. This result is consistent with Crandon-Malamud’s work on medical pluralism in the Bolivian highlands [14,44,45]. She argues that individuals select from the various medical systems available in a process of negotiation of cultural identity and access to economic, social, and political resources. In Belize, also a pluralistic setting, another study showed that individuals select from alternative therapies and etiologies consistent with their understanding of an illness in a continuing process of negotiation [46]. These studies and our own results imply that the boundaries between the various medical systems are fluid and that there may be a mutual appropriation of resources and conceptions between them [14].

The third consideration regarding households’ health-seeking strategies is that the therapeutic choices at the study sites were sometimes the outcome of power struggles between representatives of different medical systems. From this perspective, the example of labor and postpartum practices is interesting in assessing the differing influence of the formal health sector on people’ s health-seeking behavior at the study sites. While in Waca Playa these practices remain fully within the scope of Andean medicine (women give birth at home with the help of relatives and midwives, based on extensive local knowledge of delivery care and use of plants), in Pitumarca they have drastically changed in recent years as a result of the attitude and policies of the health center’s workers. Indeed, interviews with biomedical professionals showed that their attitude towards Andean medicine differed at the study sites. While the auxiliary nurse from Waca Playa recognized the importance of Andean medicine and seemed to favor possible collaboration with local healers, the staff from Pitumarca’s health center was openly dubious about healers’ and laypeople’s medical competence. One of Pitumarca medical center’s main campaigns during the last decade was to discourage the practice of home birth, in an attempt to lower maternal and infant mortality rates. Health workers based in the villages were instructed to observe and report pregnant women to the health center. Furthermore, women were fined if they did not go to the medical center for routine maternity controls and childbirth. As a consequence of these measures, most women now give birth at the health center (approximately 70% of the women from Pitumarca district, according to the health center’s physician), whereas they practiced home-birth with the help of relatives and sometimes midwives until a few years ago. Interestingly, all the women we interviewed mentioned that their husbands accompanied them to the health center and gave them medicinal herbal teas and drinks to facilitate labor, typically boiled *chicha *(a fermented drink made of maize, wheat, or other grains) and *manzanilla (Matricaria recutita* L.)*.* Furthermore, once back home, they were given herbal teas from *k’irihampi *(remedies for body pains and bruises) – for example, *thurpa (Nototriches* spp.*), chirichiri* (*Grindelia boliviana *Rusby), *yawarch’unka* (*Oenothera multicaulis* Ruiz &Pav.), or *wichullu* (undet.) - and submitted to traditional postpartum rituals and treatments, such as the *q’apachi* (incense burnt as a protection against “bad winds” and malevolent spirits) and *walt’aska* (wrapping up of the whole body with a mixture of medicinal plants and honey, usually performed by women specialists). In this example, biomedicine clearly imposes itself by coercive means upon the indigenous medical systems. However, households from Pitumarca -while apparently accepting this imposition and changing their behavior- maintained part of their own self-treatment and specialist practices from Andean medicine in a process of cultural resistance. In the Andes, as in many other parts of the world, biomedicine dominates over indigenous medical systems. According to Vandebroek et al. [13], for instance, many Bolivian doctors and nurses believe that biomedicine is superior and should replace Andean medicine. However, scholars working on medical pluralism in the Andes stress that laypeople are positively inclined towards dual use of distinct, competing, therapeutic traditions [11,15] and that “indigenous healers and medical systems remain strong, both for their perceived efficacy and expertise and for the expressions of cultural identity they represent” ([31], p. 10). Crandon-Malamud also recognizes that “resistance (…) may frequently be the objective of medical dialogue” ([14], p.-32). Our results thus show that coexisting healing traditions can be simultaneously complementary and conflicting. This is supported by the work of Ngokwey [9] in Brazil, who reported both multiple use of popular remedies and pharmaceutical drugs and differential expectations of efficacy with these two types of remedies, resulting from the perceived superiority of biomedicine in local culture.

Contrary to what other researchers have shown [5], our findings suggest that the health-seeking strategies of households in Waca Playa and Pitumarca are basically independent of the level of access to biomedicine, except for some specific practices such as childbirth. At both study sites, self-treatment with natural remedies is undoubtedly the first strategy adopted. This finding is corroborated by other studies [18,22,47]. The difference observed in the second option for health care in Waca Playa and Pitumarca can be interpreted as follows: In Waca Playa, where the availability of formal health services is limited, households consult more with indigenous healers when they cannot rely on their own knowledge and use of natural remedies to cure themselves. Conversely, in Pitumarca, where biomedical health care is not only more accessible and affordable, but also imposes itself by coercive means, households rely less on indigenous healers. One reason for this difference is that households are compelled to go to the medical post: when adding the three households that reported this situation to the six that chose freely to consult a healer, we obtain the same figures as in Waca Playa. The other reason why these households rely less on healers is not because they do not trust in their capacities, but because they do not feel as much need for their help. Elsewhere we have shown that knowledge about medicinal plants was greater in Pitumarca than in Waca Playa [35], and we suggest that this superior knowledge makes people there less dependent on the knowledge of specialists in Andean medicine.

### Use of and preference for pharmaceutical versus natural remedies

At both study sites, all 18 households used natural remedies such as plants, animals, or minerals to treat themselves. In addition, 14 also used pharmaceuticals, while the remaining 4 stated that they never used them. The results were identical in Waca Playa and Pitumarca. 

**Figure 8:**
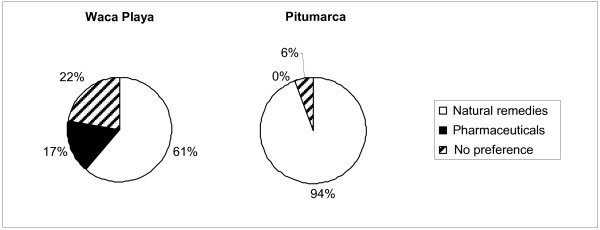
Preference for pharmaceuticals versus natural remedies for 18 households in Waca Playa (Bolivia) and 18 in Pitumarca (Peru).

The percentage of households that preferred pharmaceuticals to natural remedies at each study site is presented in Figure 8. Paradoxically, the results show that significantly more households from Pitumarca (17 households) preferred natural remedies over pharmaceuticals compared to Waca Playa (11 households) (z = 2.001; P = 0.045), where there is more limited access to formal health care services and lower indicators of human development. Only three households from Waca Playa and none from Pitumarca had a preference for pharmaceuticals.

Of the eleven households in Waca Playa that preferred natural remedies, four explained this preference by economic reasons, as pharmaceuticals were costly whereas natural remedies were free. Four said that natural remedies were more efficient in healing them. One household also mentioned that natural remedies were healthier, whereas pharmaceuticals were harmful. The three that preferred pharmaceuticals mainly used painkillers, stating that they were efficient and healed quicker than natural remedies. One household preferred pharmaceuticals owing to insufficient knowledge of natural remedies.

 Almost all households in Pitumarca preferred natural remedies. Two main arguments were mentioned to support this choice. First, plants, animals and minerals were seen as “natural” and thus healthier, whereas pharmaceuticals were “chemical” and harmful – pharmaceuticals are believed to be drugs to which the body becomes addicted and that cause other illnesses (11 households). Second, pharmaceuticals were considered less efficient than natural remedies and only able to sooth pain momentarily (8 households). Only two households mentioned that they preferred natural remedies for economic reasons. A few households and a healer from Pitumarca stated that for two or three years there had been a process of revalorization of the use of natural remedies as a consequence of increasing awareness about the beneficial properties of medicinal plants and a corresponding growing critical appraisal of biomedicine. Following is a representative statement:

"*“[People] now use more traditional medicine; they are revalorizing the properties of medicinal plants. I am working with agroecology; we are thus consuming everything natural and seldom go to the [medical] post. (…) Formerly they used to go more to the post; but (…) when I was a child they always healed themselves with plants. (…) [Nowadays] people are starting to revalorize [traditional medicine]. (…) [The post] is not recommendable; it only soothes pain and cannot heal totally.” (Pitumarca- Healer, 08/12/2008)*"

Unexpectedly, our results demonstrate that the use of pharmaceuticals or natural remedies is independent of the availability of biomedical health care in terms of quality of services provided, physical access, and affordability. Furthermore, a preference for natural remedies over pharmaceutical coexists with biomedical healthcare that is both accessible and affordable.

Our findings partially contradict the results of Vandebroek et al. [13] in the Bolivian Amazon and the Andes. Their study showed a strong negative correlation between a community’s physical isolation (and thus access to biomedicine) and the use of pharmaceuticals in the lowlands. In contrast, a similar proportion of informants used pharmaceuticals and medicinal plants in the highlands, where there was a medical post. They explained the popularity of medicinal plants at the latter site in terms of their importance in Andean culture. Even though a few participating households and people in resource-poor countries elsewhere mentioned that the cost of pharmaceuticals was one of the main reasons for preferring natural remedies [4,18], our results do not support this observation, since there was a negative association between the preference for pharmaceuticals and indicators of human development. Our results suggest that increased access to and experience with biomedicine might actually lead to a valorization of mostly local natural remedies and rejection of pharmaceuticals. While in Waca Playa a few families had more trust in pharmaceuticals, in Pitumarca there was an emerging revalorization of natural remedies. At the latter site, natural remedies can be considered elements of an indigenous medical system revalorized in response to its confrontation with a global and dominant medical system. This process is also fuelled by the concomitant strengthening of exogenous, agroecological development projects in the area that aim not only to increase the use of organic products in agriculture instead of chemicals, but also to strengthen cultural identities and local environmental knowledge as part of an integral effort to enhance biocultural diversity [48].

### Knowledge about culture-bound illnesses

The households from Waca Playa and Pitumarca mentioned a total of 74 and 93 different illnesses cured by medicinal plants, animals, or minerals, respectively. For analytical purposes, these illnesses were grouped into 50 illness categories common to the two study sites, 17 of which can be defined as “culture-bound” (CBI). These CBI categories are listed and described in Table 2, according to an *emic* perspective. During the free listing exercises, participants gave a total of 778 (Waca Playa) and 1511 (Pitumarca) different responses about medicinal use of natural remedies, respectively. Of this total, 45 (Waca Playa) and 53 (Pitumarca) were omitted and reserved for further analysis because they corresponded with illnesses that were too poorly defined, such as “serious illness that could not be healed with another remedy” or “all illnesses”. Of the remaining 743 (Waca Playa) and 1458 (Pitumarca) responses, 181 (Waca Playa) and 624 (Pitumarca) were considered CBIs in the analysis. The overall incidence per study site and detailed incidences of each CBI category in the participants’ responses are shown in Table 2.

**Table 2:** Description of culture-bound illness categories (CBIs) and their incidence in Waca Playa (Bolivia) and Pitumarca (Peru)

**Illnesses (local names in Quechua and/or Spanish)**	**Symptoms and/or etiology**	**Incidence in Waca Playa (N = 743)**	**Incidence in Pitumarca (N = 1458)**
Arrebato	Mostly experienced by women. Symptoms include headache, fever, dizziness	0.3%	0.2%
Calor interno	Internal inflammation, fever; caused by heat exposure	0.0%	1.2%
Caracter fuerte, agitación	Strong character, irritability, agitation	0.1%	0.3%
Colerina, cólico (W), cólera (P)	Stomachache, headache, bitter mouth, nauseas, vomiting; caused by anger	6.3%	1.6%
Costado	Strong side pain, hemorrhagic cough, stomachache; caused by cold exposure	0.4%	0.5%
Empacho, cólico (P)	Indigestion, stomachache, constipation; caused by ingestion of cold food	0.3%	3.2%
Enfermedad de yatiri (W), enfermedad de paqo (P)	Several illnesses cured or rituals performed by healers: possession by ancestral spirits, witchcraft, divination and diagnosis, animal sacrifice	4.6%	6.2%
Enfriamiento, enfermedad del frio	Stomachache, headache, body pain, cold feet; caused by cold exposure	0.0%	0.7%
Japega (W), mancharisqa (P), susto	Mostly experienced by children. “Fright sickness”:’ insomnia, continuous crying; caused by fall in an evil place, fright, and soul loss	1.6%	0.5%
Madre, erita (P), cáncer (P)	Experienced by women. Symptoms of uterine or ovary inflammation, hot body, peeling skin, swollen hands and feet, back pain; caused by heat or cold exposure during menstruation	2.0%	7.0%
Mal de bilis, hígado	“Biliary sickness”: nausea, dry and acid taste in mouth, liver inflammation; caused by ingestion of greasy food, also caused by anger	0.3%	1.6%
Mal de corazón, perdida de memoria	“Heart sickness”: headache, dizziness, memory loss; caused by worry or sadness	0.1%	1.9%
Protección espiritual, suerte	Mostly used for children. Spiritual protection against "bad winds" such as *soqawayra* or *machuwayra,* amulet for good luck	0.1%	0.8%
Rayo (W), qhaha (P)	Hemorrhagic cough, weight loss, paralysis of the arms; caused by lightening	0.5%	3.9%
Tuku (W), tukuchi (P), ataques	Attack, epileptic fit, dizziness, weakness, fainting; caused by *mal de corazón*	0.9%	1.4%
Wayra, malviento (uraña, soqa, machu, etc.)	Several types of “bad winds” (*uraña, soqawayra, machuwayra*, etc.): vomiting, headache, stomachache, sore feet, paleness, weakness, cold body, paralysis, inability to speak, can lead to death; caused by encounters with animals or spirits when one walks at night, also caused by anger	5.5%	11.5%
*Other culture-bound illnesses* (oreja, cuyca, chiripa, phasku)	*Oreja* (mostly in children): weight loss, diarrhea; caused by the smell of a dead animal or human body		
	*Cuyca:* internal illness		
	*Chiripa*: caused by rainbow		
	*Phasku*: (mostly in babies) dry and cracked lips and skin; caused by exposure to sun.		
**Total incidence of CBIs**		**24.4%**	**42.8%**

Illnesses: local names are followed by (W) when used only in Waca Playa and (P) when used only in Pitumarca. Incidence: percentage of the participants’ total responses about natural remedies’ medicinal uses.

There was a significant difference between the incidence of CBIs in Waca Playa (24.4%) and Pitumarca (42.8%) (z = 8.428; P = <0.001). This does not necessarily mean that there was a higher occurrence of CBIs in Pitumarca, but it implies greater knowledge about CBIs, their symptoms and etiology at the latter site. It may also indicate that Pitumarquinos have deeper Andean medical knowledge and understanding of all illnesses in general and of their relationships with the various spheres of Andean cosmology, including natural, social, and spiritual domains. For example, an ailment simply described as “headache” in Waca Playa (not classified as CBI), was described as “headache and dizziness caused by exposure to *urañawayra* during a night walk” in Pitumarca (classified as CBI). A condition expressed as “stomach ache” in Waca Playa would be depicted as “*cólera* or stomach ache and bitterness transmitted through breastfeeding a baby by a mother who is angry due to a recent fight with a close relative.” The higher incidence of CBIs in responses from households in Pitumarca could also indicate a stronger affirmation of cultural identity, corresponding to Greenway’s results [41]. She interprets some cases of fright sickness and their healing ceremonies in the Southern Peruvian Andes as a process of “resistance to submersion in a wider national identity.” ([41], p. 1003).

## Conclusions

We believe our work makes an important contribution to the body of empirical ethnomedical research by de-monstrating that the increased presence of the formal health sector does not necessarily displace indigenous medicine in the rural Andes, but that these two systems can coexist in complex ways. Three interesting insights about Andean households’ health-seeking behavior can be derived from this study. First, the health strategies of Andean households denote a strong aspiration for autonomy in the management of health based on own cultural beliefs. This was observed in the prevalence of self-treatment over other therapeutic strategies and in valorization of own knowledge about natural remedies. This striving for autonomy echoes current struggles of Andean peasants in other spheres of daily life, such as in governance and management of territory and natural resources [49]. Second, Andean households are aware of the limitations of the formal health system in responding to CBIs, and of the potential contributions of biomedicine in treating other ailments with mainly physical origins. This leads them to a pragmatic, self-determined search for complementarities between different medical systems, reflected in their therapeutic itineraries. Third, the encounter between these various medical ideologies, far from being harmonious, is characterized by the tentative hegemony of biomedicine over indigenous medical traditions. This leads to changes in some health practices in Andean households but at the same time to a reflexive maintenance and revalorization of other aspects of indigenous medicine, in a process of cultural resistance and renewal. Ethnomedicine thus becomes a resource for cultural affirmation in its confrontation with the dominant medical system. More studies are required to determine whether these findings apply to other settings characterized by medical pluralism in rural areas of the developing world.

The take-home lesson for health policy-makers is that the main obstacle to use of biomedicine in resource-poor rural areas might not be infrastructural or economic alone. It may lie in a lack of sufficient recognition by biomedical practitioners of the value and importance of indigenous medical systems. We propose that implementation of health care in indigenous communities be designed as a process of joint development of complementary knowledge and practices from indigenous and biomedical health traditions. This could be achieved through a social learning process that explores complementarities between biomedicine and indigenous medical systems based on dialogue, cultural sensitivity, and equitable participation of representatives from existing health traditions.

## Competing interests

The authors declare that they have no competing interests.

## Authors’ contributions

SLMS conceived and designed the study, collected and analyzed the data, and drafted the manuscript. IV participated in the data analysis. SR helped with the design of the study. All authors contributed to the interpretation of the data and revision of the manuscript. All authors approved the final version of the manuscript. Photographs were taken by SLMS.
